# Graphitic Carbon Nitride: A Highly Electroactive Nanomaterial for Environmental and Clinical Sensing

**DOI:** 10.3390/s20205743

**Published:** 2020-10-10

**Authors:** Azeez O. Idris, Ekemena O. Oseghe, Titus A. M. Msagati, Alex T. Kuvarega, Usisipho Feleni, Bhekie Mamba

**Affiliations:** Institute for Nanotechnology and Water Sustainability (iNanoWS), Florida Campus, College of Science, Engineering and Technology, University of South Africa, Johannesburg 1709, South Africa; osegheo@unisa.ac.za (E.O.O.); msagatam@unisa.ac.za (T.A.M.M.); atkuvarega@unisa.ac.za (A.T.K.); felenu@unisa.ac.za (U.F.); mambabb@unisa.ac.za (B.M.)

**Keywords:** graphitic carbon nitride (g-C_3_N_4_), heavy metals, biosensors, electrochemical sensors, nanoparticles

## Abstract

Graphitic carbon nitride (g-C_3_N_4_) is a two-dimensional conjugated polymer that has attracted the interest of researchers and industrial communities owing to its outstanding analytical merits such as low-cost synthesis, high stability, unique electronic properties, catalytic ability, high quantum yield, nontoxicity, metal-free, low bandgap energy, and electron-rich properties. Notably, graphitic carbon nitride (g-C_3_N_4_) is the most stable allotrope of carbon nitrides. It has been explored in various analytical fields due to its excellent biocompatibility properties, including ease of surface functionalization and hydrogen-bonding. Graphitic carbon nitride (g-C_3_N_4_) acts as a nanomediator and serves as an immobilization layer to detect various biomolecules. Numerous reports have been presented in the literature on applying graphitic carbon nitride (g-C_3_N_4_) for the construction of electrochemical sensors and biosensors. Different electrochemical techniques such as cyclic voltammetry, electrochemiluminescence, electrochemical impedance spectroscopy, square wave anodic stripping voltammetry, and amperometry techniques have been extensively used for the detection of biologic molecules and heavy metals, with high sensitivity and good selectivity. For this reason, the leading drive of this review is to stress the importance of employing graphitic carbon nitride (g-C_3_N_4_) for the fabrication of electrochemical sensors and biosensors.

## 1. Introduction

Graphitic carbon nitride (g-C_3_N_4_) is a two-dimensional conjugated polymer consisting of carbon and nitrogen. It is obtained from different carbon materials analogues by replacing carbon atoms with nitrogen atoms [1]. Also, graphitic carbon nitride (g-C_3_N_4_) is one of the oldest documented polymers in the literature with the general formula (C_3_N_3_H)_n_; the history of this material can be dated as far back as 1834 [2]. According to the literature, the carbon nitride (CN) material was first studied in 1834 when Berzelius obtained a linear CN polymer and named it “melon” [2]. This breakthrough led to the discovery of graphitic carbon nitride (g-C_3_N_4_), prepared by thermal decomposition of mercuric thiocyanate by Franklin in 1922 [3]. In 1989, numerous studies indicated that if carbon could replace silicon in the structure of silicon nitride (β-Si_3_N_4_), a very hard carbon nitride (β-C_3_N_4_) may be obtained [3].

Furthermore, in 1966, Teter and Hemley predicted various phases of several allotropic forms of carbon nitride, which include α-C_3_N_4_, β-C_3_N_4_, cubic-C_3_N_4_, pseudo-C_3_N_4,_ and graphitic-C_3_N_4_ [3]. It was reported that all the phases of the CN materials are super-hard except g-C_3_N_4,_ and it was documented to be the most stable allotrope under ambient conditions [3]. This report revealed the ease of modifying the structure and the morphology of graphitic carbon nitride. As a result, the chemistry of different morphologies, structures, and graphitic carbon nitride (g-C_3_N_4_) applications has attracted considerable interest from scientists and industrial communities in employing this material for various analytical applications.

The carbon and hydrogen atoms in graphitic carbon nitride (g-C_3_N_4_) are bonded together by sp^2^ hybridization. In graphitic carbon nitride (g-C_3_N_4_), both carbon and nitrogen atoms are sp^2^ hybridized and are connected by σ bonds forming a hexagonal structure. This structure is called a triazine ring, and each of these rings is linked to a small unit connected by a C–N bond. There is a consensus that graphitic carbon may have two chemical structures, namely g-C_3_N_4_ with a triazine ring (C_3_ N_3_), which belongs to the *R3 m* space group, and the other structure consists of the tri-s-triazine ring (C_6_N_7_) [4]. In graphitic carbon nitride (g-C_3_N_4,_) each triazine ring is connected by the nitrogen atom at the end, forming a noticeably expanded planar grid structure.

**Figure 1:**
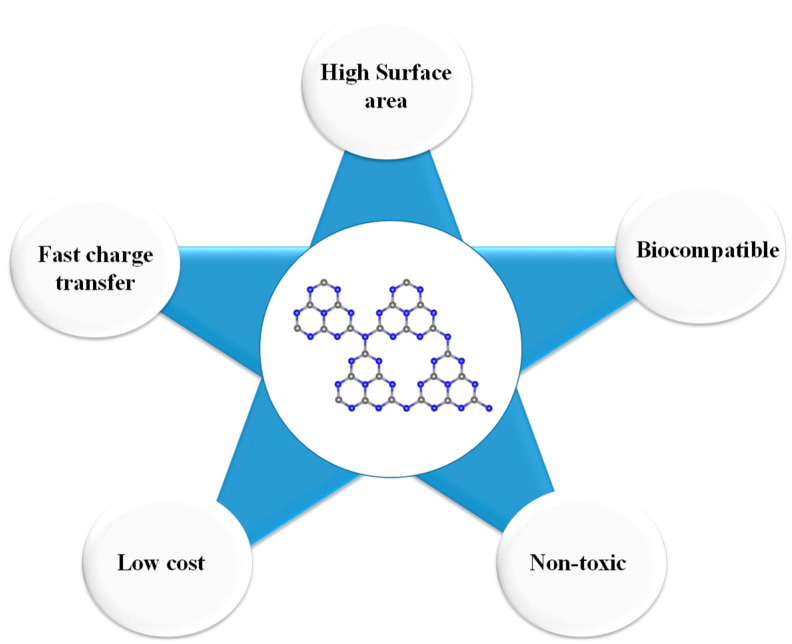
Critical properties of graphitic carbon nitride (g-C_3_N_4_) that promote its application for sensor fabrication.

Graphitic carbon nitride (g-C_3_N_4_) has recently fascinated researchers’ interest due to its outstanding properties, including low-cost, large surface area, earth-abundant, fast electron transfer π-π conjugation structure, metal-free, excellent visible-light-driven polymeric semiconductor, biocompatibility, and catalytic properties [5,6]. Figure 1 depicts graphitic carbon nitride’s key features that spurred scientists towards employing graphitic carbon nitride (g-C_3_N_4_) for various analytical fields.

It is important to emphasize that the excellent catalytic and biocompatibility properties of graphitic carbon nitride (g-C_3_N_4_) have inspired various scientists to utilize this material to construct multiple sensors and bio-(sensors). Thus, this review aims to provide a comprehensive assessment of the synthesis and application of graphitic carbon nitride (g-C_3_N_4_) to construct sensors and biosensors.

## 2. Preparation of Graphitic Carbon Nitride (g-C_3_N_4_)

Several synthesis routes have been reportedly used to prepare graphitic carbon nitride (g-C_3_N_4_), taking cognizance of the relationship between the structure of the material and morphologic features. In summary, top-down and bottom-up approaches are the synthesis routes that are generally employed to synthesize various morphologies of graphitic carbon nitride (g-C_3_N_4_) [6]. The former involves liquid and thermal exfoliation methods, while the latter involves a solvothermal approach, supramolecular aggregation, and soft and hard templates [6]. The top-down synthesis route involves the sequential breaking down of larger blocks of graphitic carbon nitride (g-C_3_N_4_) into smaller units, g-C_3_N_4_ nanosheets multilayer, and single-layer structures. Similarly, the bottom-up approach involves the incorporation of smaller molecules to form more complex molecules. In contrast, the bottom-up approach is more advantageous than the top-down approach because the former has a better chance of producing nanostructures with fewer defects, more homogenous chemical composition, better short- and long-range ordering.

Generally, there are four principal ways of preparing graphitic carbon nitride (g-C_3_N_4_): (1) sol-gel method, (2) soft-template method, (3) hard-template method, and (4) template-free method [7]. While the sol-gel and thermal polymerization method can be classified as a bottom-up approach, ultrasound-assisted exfoliation and template-assisted method can be classified as a top-down approach.

It is essential to highlight that these methods employ different methodologies and precursors, which invariably lead to products with different morphologies, sizes, and properties. A brief discussion of the various synthesis routes is discussed in the next section.

### 2.1. Sol-Gel Method

The sol-gel method is one of the most widely used techniques for the synthesis of nanomaterials due to its simplicity and efficient and sustainable model for bulk synthesis of nanomaterials. This approach’s beauty is that it allows controlled growth and tailored design of the nanomaterial pore structure. This approach involves mixing the precursor of graphitic carbon nitride (g-C_3_N_4_) with the precursor of silica. Subsequently, the graphitic carbon nitride (g-C_3_N_4_) is finally obtained after several successive steps, including sequential sol mixing, gel formation, calcination of the gel, and silica removal [8].

For instance, Yang et al. prepared mesoporous graphite carbon nitrides by magnetically stirring a mixture of amino cyanide and colloidal silica solution for 5 h. After that, the resulting homogeneous and viscous solution was dried at 70 °C for 60 min to obtain a transparent gel [8]. The amino cyanide used as a precursor for this synthesis is very toxic; therefore, we recommend eco-friendly precursor compounds for the synthesis of graphitic carbon nitride (g-C_3_N_4_). Furthermore, the gel was calcined at 550 °C for 4 h under nitrogen. A dark yellow substance was obtained and treated with 4 M NH_4_HF_2_ solution to remove the silica template. Finally, a yellow powdery solid was harvested and washed with copious deionized water and ethanol. [8]. Besides, in our view, the NH_4_HF_2_ solution employed in removing the silica template is very toxic. It can react with the calcium in the body if not carefully used and handled. Hence, we discouraged applying this poisonous compound for the preparation of graphitic carbon nitride (g-C_3_N_4_). The reaction that may occur if NH_4_HF_2_ is accidentally ingested into the body is depicted in Equation (1).

NH_4_HF_2(aq)_ + Ca(s) → CaF2(s) + NH_3_(g) + H_2_(g) (1)

### 2.2. Ultrasound-Assisted Exfoliation Method

This approach helps increase the phase purity of graphitic carbon nitride powder, decrease its energy band gap, change the morphology, and enhance the material’s specific surface area. For this reason, Chebanenko et al. successfully prepared graphitic carbon nitride (g-C_3_N_4_) by heating 10 g of urea at 550 °C in a quartz tube for 2 h; the resulting material was removed from the tube after cooling [9]. Subsequently, the material was added to distilled water and stirred for a few minutes with a magnetic stirrer’s aid. The resulting white suspension was exposed to an ultrasonic disintegrator for 2 h. Consequently, the colloidal solution was dried in an oven at 100 °C to remove water molecules and annealed at 300 °C for half an hour to obtain the dry exfoliated g-C_3_N_4_ nanopowder [9].

**Figure 2:**
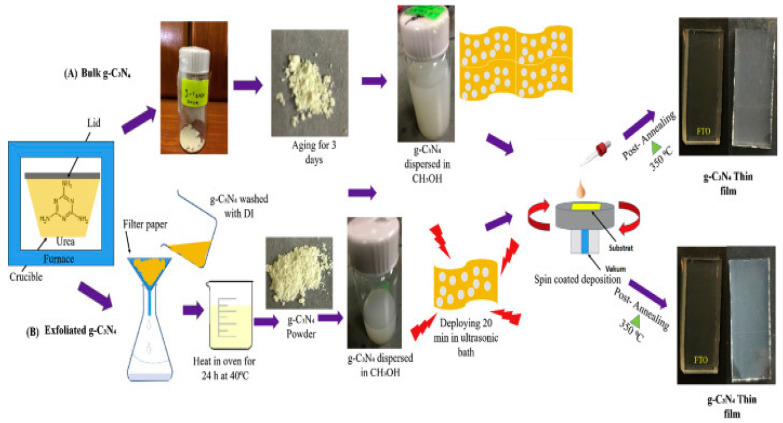
Schematic illustration of sample preparation and fabrication of (**A**) bulk and (**B**) exfoliated graphitic carbon nitride (g-C_3_N_4_) thin film. (Copyright 2020 Elsevier; used with permission) [10].

On the other hand, the synthesis of bulk graphitic carbon nitride (g-C_3_N_4_) was done by heating urea at 520 °C for 30 min and cooled in the furnace at 100 °C. Hereafter, it was dispersed in methanol and kept in a glass bottle for three days [10]. The exfoliated graphitic carbon nitride (g-C_3_N_4_) was prepared by washing the bulk graphitic carbon nitride (g-C_3_N_4_) with deionized water via the filtration process. Afterward, the graphitic carbon nitride (g-C_3_N_4_) was heated in an oven at 40 °C overnight, and the exfoliated g-C_3_N_4_ powder obtained was dispersed in methanol and ultrasonicated for 20 min. A significant problem associated with urea as a precursor for the preparation of graphitic carbon nitride (C_3_N_4_) is the pungent smell that emanated from the furnace during synthesis. We recommend that this experiment be performed in a fume cupboard to trap the gases oozing out from the reaction mixture during synthesis due to the promising synthesis results. The scheme for the synthesis of exfoliated graphitic carbon nitride (g-C_3_N_4_) is presented in Figure 2.

### 2.3. Template-Assisted Method

This method employs organic or inorganic molecules for the synthesis of porous graphitic carbon nitride (g-C_3_N_4_). Herein, block polymers and mesoporous silica are used to prepare mesoporous carbon materials, including graphitic carbon nitride (g-C_3_N_4_). For instance, Wang et al. employed silica nanoparticles to prepare mesoporous graphitic carbon nitride (g-C_3_N_4_) using cyanamide’s thermal condensation [11]. In our assessment, cyanamide’s apparent toxicity is a significant disadvantage of the synthetic route employed for the synthesis of graphitic carbon nitride (g-C_3_N_4_) by Wang et al.

### 2.4. Thermal Polymerization Method

This method involves the conversion of monomer to polymer using thermal energy. There are two types of thermal polymerization, which are homo- or co-polymerization. The former requires a substantial amount of thermal energy while the latter occurs at ambient temperature between electron–acceptor, and electron–donor monomer. For instance, Zhao et al. prepared graphitic carbon nitride (g-C_3_N_4_) from various precursors (dicyandiamide, melamine, and guanidine carbonate) via fractional thermal polymerization method [12]. It is crucial to point out that the graphitic carbon nitride (g-C_3_N_4_) obtained from different precursors has a different bandgap, morphology, and photocatalytic activities/ability [12]. Summarily, the synthesis of graphitic carbon nitride (g-C_3_N_4_) has been reported in the literature [13,14,15]_._

## 3. Multifunctional Applications of Graphitic Carbon Nitride (g-C_3_N_4_)

Graphitic carbon nitride (g-C_3_N_4_) is attracting enormous research interest because it is the most stable allotrope of carbon nitride coupled with some exciting analytical merits, including low-cost synthesis, biocompatibility, high water dispersibility, photocatalytic properties, unique electronic properties, and nontoxicity [16,17,18]. These outstanding properties prompted scientific communities to explore graphitic carbon nitride (g-C_3_N_4_) for various applications, including sensing heavy metals, organic pollutants, biosensors, gas sensing, and quantification pharmaceuticals in water.

The application of graphitic carbon nitride (g-C_3_N_4_) for sensor development is discussed in the next section.

### 3.1. Sensing of Metal Ions Using Graphitic Carbon Nitride (g-C_3_N_4_)

#### 3.1.1. Pb^2+^

As mentioned earlier, several reports on the application of polymeric graphitic carbon nitride (g-C_3_N_4_) for quantification of heavy metals are attributed to the excellent and remarkable chemical stability, low cost, and non-toxic properties of graphitic carbon nitride (g-C_3_N_4_) [16,17,18]. Consequently, a two-dimensional graphitic carbon nitride (g-C_3_N_4_) was prepared via the liquid exfoliation method. The graphitic carbon nitride was used to modify a glassy carbon electrode (GCE) for fast and ultra-sensitive voltammetry detection of Pb^2+^ in water [19]. The detection limit of 1 ng/mL was reported, and the sensor was highly selective in the presence of common ions (K^+^, Na^+^, Ca^2+^, Mg^2+^, NO_3_^−^, Cu^2+^, Zn^2+,^ and Fe^2+^) that compete with the ion of interest (Pb^2+^) in the solution. Thus, the fabricated sensor was used to quantify Pb^2+^ in drinking water and urban street dust [19]. It was reported that the free electron pairs (nitrogen atoms) in the graphitic carbon nitride acted as the active sites for the adsorption of Pb^2+^ on the electrode surface. As a result, the electrochemical sensor for Pb^2+^ displayed high sensitivity. We recommend that Nafion should be used as a stabilizing agent during sensor fabrication to prevent the leaching of the prepared graphitic carbon nitride or nanomaterials inside the electroanalytical solution.

Furthermore, Zou et al. doped graphitic carbon nitride (g-C_3_N_4_) with sulfur to increase the intrinsic active centers, enhance the electron transfer kinetics, and its electrochemical performance for the detection of Pb^2+^. However, various heteroatoms such as oxygen, boron, phosphorus, and sulfur can be used for doping [20]. Yet, sulfur was selected due to the strong affinity of lead to sulfur, which helps to attract the migration of Pb^2+^ to the surface of the sulfur-doped graphitic carbon nitride (g-C_3_N_4_) nanoflakes, a detection limit of 3 × 10^−9^ mol/L was obtained [20]. The sensor’s remarkable performance was attributed to the short bond length of S–Pb, which initiated strong interaction between positive and negative Milliken charges at Pb and S atoms. The equation for sulfur’s affinity to lead in this report is shown in Equation (2).

Pb(s) +S(s) → PbS(s)
(2)

**Figure 3:**
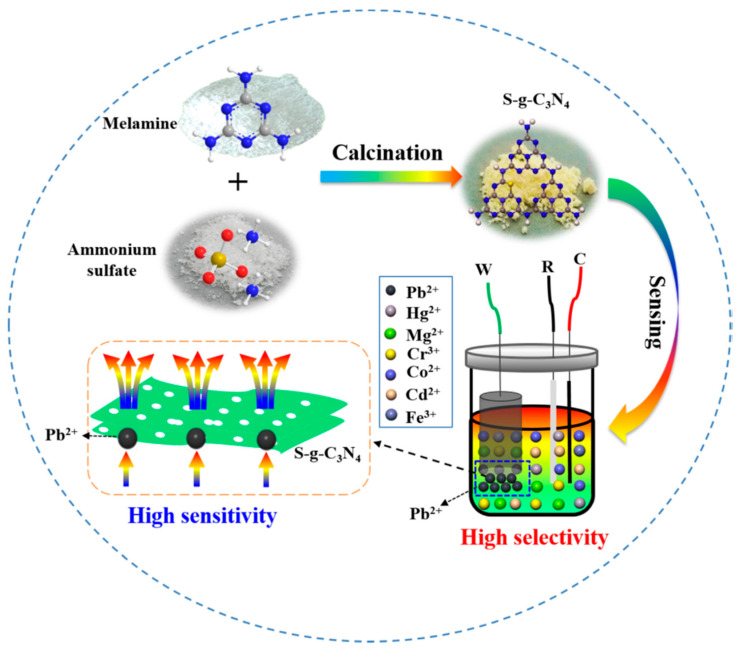
Schematic of the electrochemical detection of Pb^2+^ using sulfur-doped graphitic carbon nitride (g-C_3_N_4_) (Copyright 2019 Elsevier; used with permission) [20].

The scheme for preparing graphitic carbon nitride (g-C_3_N_4_) and electrochemical detection of Pb^2+^ using sulfur-doped graphitic carbon nitride (g-C_3_N_4_) is presented in Figure 3.

In addition to this, graphitic carbon nitride (g-C_3_N_4_), in conjunction with bismuth films, was used to develop a Pb^2+^ sensor with a detection limit of 2 pM [21]. The sensor’s remarkable performance was due to the sp2-bonded carbon and nitrogen atoms with surplus π electrons in the layers, which helped attract the analyte on the electrode surface. Also, the ability of Pb^2+^ to coordinate with several N-atoms of graphitic carbon nitride or enters its layers during accumulations. As reported, bismuth films can be deposited on a conducting substrate either by an electrochemical deposition method, i.e., by applying constant potential either in-situ (in the model or sample solution) or ex-situ by pre-deposition [21]. In this case, Bi-film was incorporated into graphitic carbon nitride (g-C_3_N_4_) by the electrodeposition method. However, in our previous study, Bi-film was deposited on a conducting substrate (exfoliated graphite electrode) for the simultaneous detection of notorious heavy metals (As, Hg and Pb) [22]. In this regard, we recommend the application of an exfoliated graphite electrode for various electrochemical applications. In our assessment, this report’s beauty is that the bismuth film prevented the leaching of the graphitic carbon nitride (g-C_3_N_4_) into the analyte solution during sensing. However, the interaction between bismuth and graphitic carbon nitride (g-C_3_N_4_) should be investigated.

In another study, a novel graphitic carbon nitride (g-C_3_N_4_) nanocone (g-CNNC) was prepared and employed in the construction of a fluorescent sensor for the detection of Pb^2+^ [23]. The results showed that the g-CNNC displayed impressive selectivity and sensitivity for fluorescence quantification of Pb^2+^ via covalent interaction [23]. The FTIR result confirmed that the Pb^2+^ ion bonded to the terminal amino group, C–N, and C=N heterocycles of g-CNNCs via covalent interaction. Hence, a lower detection limit was obtained. It is essential to highlight that fluorescent sensors rely predominantly on fluorescence quenching interactions between the nanomaterials and the analyte of interest [23]. The fluorescence technique is one of the most important analytical methods for detecting various substances at trace levels due to fast response time, low-cost, high sensitivity, and selectivity [23].

Furthermore, Zhang et al. fabricated an electrochemiluminescence (ECL) sensor to monitor Pb^2+^ in environmental samples by decorating graphitic carbon nitride (g-C_3_N_4_) quantum dots (QDs) on reduced graphene oxide (rGO) using target-dependent DNAzyme as the recognition unit [24]. A one-pot in situ reduction approach was used to decorate Fe_3_O_4_ nanoparticles on reduced graphene oxide nanosheets (rGO-Fe_3_O_4_) as carriers for target-dependent DNAzyme [24]. The magnetic nanocomposite was immobilized on indium tin oxide (ITO) glass to detect Pb^2+^ [24]. The sensor’s high selectivity and good selectivity were attributed to the exceptional separation and enrichment properties of the nanocomposite (rGO-Fe_3_O_4_) and the wire-like conductivity of the graphitic carbon nitride. Notably, a one-step synthesis route was used in the preparation of this ternary nanocomposite. This study demonstrated the potential of using rGO for the sensing of heavy metals [24]. The advantages of using electrochemiluminescence (ECL) techniques include high sensitivity, low background signal, impressive time/spatial controllability, wide dynamic range, and a simplified optical setup [25].

In another report, graphitic carbon nitride (g-C_3_N_4_) was chemically modified with amino-calixarene as an indicator molecule via microwave irradiation and used to modify glassy carbon electrode (dia. = 5 mm) for the simultaneous detection of Pb^2+^ and Cd^2+^ at pico-molar range [26]. Interestingly, the developed sensor was successfully used in quantifying lead and cadmium ions in alloy materials, sewage sample matrices, and battery industry wastewater samples. The results obtained were concordant with the results reported for standard protocols [26]. Similarly, poly(2,5-bis(3,4-ethylenedioxythienyl) pyridine/graphitic carbon nitride (g-C_3_N_4_) composites were prepared by in situ chemical polymerization. The nanocomposites were used to modify a glassy carbon electrode for the co-detection of Pb^2+^ and Cd^2+^ using differential pulse voltammetry (DPV) [27]. The detection limit values of 0.00324 µM and 0.018 µM were reported for Pb^2+^ and Cd^2+^, respectively [27]. The sensor was stable due to the in-situ modification route employed in constructing it; this method is far better than the common drop-drying and drop-coating methods used to modify conducting substrate.

The approach employed by Adarakatti et al. focuses on the preparation of Mn_3_O_4_ nanoparticles (Mns)/graphitic carbon nitride (g-C_3_N_4_) by decomposition of sucrose–nitrate [26]. The Mns/g-C_3_N_4_ was immobilized on a glassy carbon electrode for the simultaneous detection of Pb^2+^, Cd^2+,^ and Hg^2+^ in aqueous media via differential pulse anodic stripping voltammetry (DPASV) [26]. The analytes’ detection limits were 9.66 × 10^−11^, 0.48 × 10^−11^, and 0.51 × 10^−11^ M for Pb^2+^, Cd^2+,^ and Hg^2+^, respectively. The sensor’s electrochemical activity was attributed to the highly negative surface charge of the manganese oxides, which increases the adsorption capacity of the analyte on the Mn_3_O_4_ nanoparticles. In this study, the developed sensor’s practical application was interrogated using a battery, chrome-plating, and industrial effluent samples [26]. For further improvements, better detection limits would have been obtained if square wave anodic stripping voltammetry (SWASV) was used for the simultaneous detection of the analytes because it is more sensitive than the DPASV.

**Figure 4:**
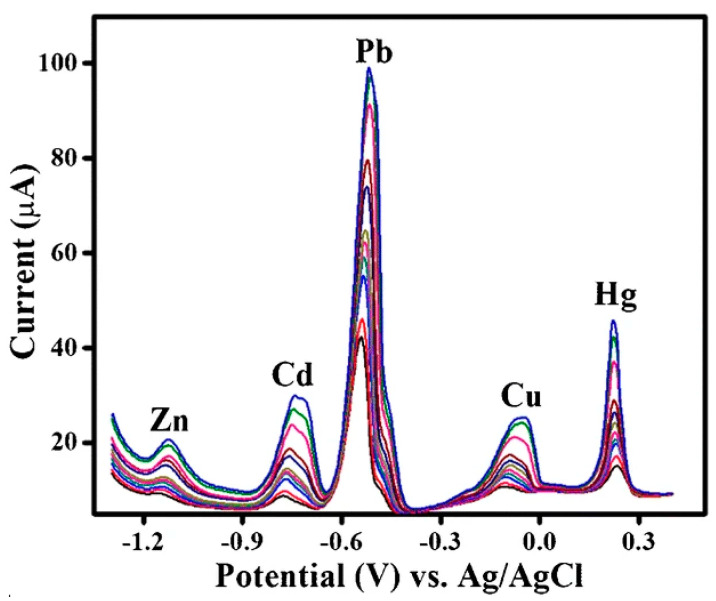
Simultaneous detection of Zn, Cd, Pb, and Hg (Copyright 2019 Springer; used with permission) [28].

Researchers have improved by introducing oxidized multi-wall carbon nanotubes (O–MWCNTs) on a 3D porous graphitic carbon nitride (g-C_3_N_4_). The nanocomposite was modified on a screen-printed electrode (SPE) for the simultaneous detection of Pb^2+^, Cd^2+^, Zn^2+,^ and Hg^2+^ [28]. Different electrochemical techniques such as cyclic voltammetry, electrochemical impedance spectroscopy, and differential pulse voltammetry were used to interrogate the quantification of these analytes. Therefore, these analytes’ simultaneous detection was possible because of well-separated peaks potentials of 0.5, −0.78, −11.6, and +0.35 V (vs. Ag/AgCl). The analytes’ detection limits were between 8 to 60 ng/L, and the simultaneous detection of these ions was also carried out in various spiked food samples [28]. Based on the obtained results, this sensor is highly recommendable owing to the conducting substrate used for the analysis. The conducting substrate was also not dipped inside the analyte solution; instead, both the modifiers and the analytes were meticulously dropped on the conducting substrate. Hence, the platform played a significant role in preventing leaching of the nanomaterials into the analyte solution. The simultaneous detection of the analytes is depicted in Figure 4.

#### 3.1.2. Detection of Hg^2+^ and Other Metals

Mercury (Hg) is one of the notorious heavy metals on the earth’s surface and extremely harmful to humans and animals, even at low concentrations [29]. Exposure to mercury or its compounds (particularly methylmercury) can cause a series of toxicological effects such as kidney failure, brain damage, deafness, vision impairment, loss of sensation, and poor muscle coordination [28,29]. It is essential to develop a fast and user-friendly sensor for Hg^2+^ ions with low cost, high sensitivity, and selectivity. For instance, Abdolmohammad-Zadeh and Rahimpour developed a novel chemosensor based on graphitic carbon nitride quantum dots (g-CNQDs) using potassium ferricyanide (K_3_Fe(CN)_6_) as an oxidant in the chemiluminescence (CL) system for the detection of Hg^2+^ ions [30]. The developed chemosensor (g-CNQDs–K_3_Fe(CN)_6_ CL system) was successfully applied for the detection of Hg^2+^ ions in water and food samples with recoveries in the range of 95.5–102.8% for spiked samples [30].

In another study, chitosan-functionalized gold nanoparticles were assembled on sulfur-doped graphitic carbon nitride (g-C_3_N_4_) for the colorimetric detection of trace Hg^2+^ [31]. The results showed that the chitosan acted as both a reducing and stabilizing agent. At the same time, graphitic carbon nitride (g-C_3_N_4_) played a significant role in synergistic interconstituent interaction this helps to increase the sensitivity and selectivity of the sensor. On the other hand, gold nanoparticles enhanced the sensor conductivity [31]. Each of the modifiers contributed significantly during the detection of the analyte. However, we recommend that the interaction and the chemistry that occurs when multiple modifiers are used during sensor fabrication should be thoroughly studied and investigated. In 2015, we published a report on selenium’s electroanalysis in water on an electrodeposited gold nanoparticle-modified glassy carbon electrode [32]. In 2017, we developed sensors to detect selenium in water using gold nanoparticles [33,34]. 

A facile approach was used for a one-step synthesis of graphitic carbon nitride nanosheets using melamine/sodium citrate, which was used to construct a label-free fluorescence quenching sensor for sensitive and selective quantification of Hg^2+^ [35]. The results revealed a linear relationship between the fluorescence intensity and the concentration of Hg^2+^ from 0.001 to 1.0 µM with a detection limit of 0.3 nM. The fabricated sensor was used in the detection of Hg^2+^ in water and milk samples [35].

Graphitic carbon nitride (g-C_3_N_4_) was used in the assembly of a fluorescence sensor to detect mercury ions in tap water samples, and a detection limit of 0.14 µM was obtained [36]. In this report, the graphitic carbon nitride (g-C_3_N_4_) was prepared by the microwave synthesis route [36]. More so, one-pot synthesis of hollow cross-linked fluorescent graphitic carbon nitride (g-C_3_N_4_) was used in the detection of Hg^2+^ [37]. The developed sensor displayed two linear relationships between fluorescence intensity and the concentration of Hg^2+^ in the range of 0.1–8 µM and 8–32 µM [37]. The limit of detection (LOD) for the Hg^2+^ was found to be 0.094 µM, and the sensor was used to quantify Hg^2+^ in tap water samples [37].

Another interesting study on a “turn-off” fluorescence biosensor based on graphitic carbon nitride (g-C_3_N_4_) was reported for the detection of Hg^2+^ [38]. In comparison, this biosensor system was fabricated by the functionalization of graphitic carbon nitride (g-C_3_N_4_) with single-stranded DNA (ssDNA) aptamer. The sensor was reported to have good selectivity with a detection limit of 0.17 nM. The g-C_3_N_4_-based fluorescence sensor was said to be a promising tool for the detection of metal ions in real samples [38]. Moreover, an ion-imprinted polymer, in conjunction with graphitic carbon nitride (g-C_3_N_4_) was used to develop a highly sensitive and selective electrochemical sensor to detect Hg^2+^ [39]. The imprinted polymer alongside graphitic carbon nitride (g-C_3_N_4_) was used to modify a carbon paste electrode (CPE) for the detection of Hg^2+^ ions, and a detection limit of 18 pM was reported [39]. The major drawback of this sensor lies in the carbon paste electrode used for sensor fabrication. This electrode suffers from the reproducibility of results during analysis because of human error during sensor fabrication. Besides this, it is very tedious and time-consuming to fill the modifiers into the CPE’s small diameter tube.

As mentioned earlier, graphitic carbon nitride (g-C_3_N_4_) has been employed as nanowire to develop various sensors due to its excellent analytical features such as optical properties, high stability, biocompatibility, and low cost [4,5]. Several studies have indicated that graphitic carbon nitride (g-C_3_N_4_) can be used to detect metal ions [40]. For example, a graphitic carbon nitride nanosheet was prepared by treating melamine powder with an acid. This material was used to modify a carbon paste electrode for the electrochemical detection of Ag^2+^ [40]. The fabricated sensor was employed to detect trace levels of silver ions in water; it was found to be highly selective and resistant against inferring cations (Hg^2+^, Cd^2+^, Cu^2+^, Pb^2+,^ and Cr^3+^); the spectroscopic technique was used to interrogate the interaction of Ag^2+^ and graphitic carbon nitride (g-C_3_N_4_) [40]. The result revealed that the silver ion had the strongest interaction with g-C_3_N_4_ than other cations [40]. Even though the electrodeposition method is the best modification route used to modify an electrode, it is practically impossible to use this route to modify CPE. Hence, a fundamental disadvantage in the application of this electrode for electrochemical analysis.

Liu et al. compared a 3D bulk material to a 2D layered material after activation treatments; the results showed that the 3D bulk material displayed superior electrochemical performance to detect Cd^2+^ [41]. Besides, a 3D branched crystalline carbon nitride was exploited to develop a photoelectrochemical (PEC) sensor to detect trace Cu^2+^ [42]. Here, the graphitic carbon nitride (g-C_3_N_4_) was dispersed on the surface of fluorine-doped titanium oxide (FTO) to form a photoanode, which was used in the photoelectrochemical detection of Cu^2+^ in an aqueous environment. We recommend incorporating metal nanoparticles into the matrix of graphitic carbon nitride (g-C_3_N_4_) to improve its photocatalytic ability. The metal nanoparticles helped to suppress photogenerated electron-hole recombination. The philosophy of photoelectrochemical sensor is the generation of photocurrent and the excitation of photoactive materials by photo-induced charge transfer. Besides, light is the excitation source used in the photoelectrochemical (PEC) system, and the generated PEC current or voltage is used as the detection signal. The PEC systems possess superior properties, including high sensitivity and low background noise [42].

In another report, graphitic carbon nitride (g-C_3_N_4_) hybridized with graphene oxide (GO) (g-C_3_N_4_/GO hybrid) was used in the development of an electrochemiluminescence (ECL) sensor for the ultrasensitive detection of Cu^2+^ in water [43]. From the findings, the GO did not only assist in enhancing the cathodic ECL signal, but also for the immobilization of graphitic carbon nitride (g-C_3_N_4_). The sensor was used to detect Cu^2+^ in real wastewater samples and was recommended as a promising alternative method for routine quantification of Cu^2+^ in real wastewater samples [43]. An improved detection limit of the analyte would have been obtained if the GO were chemically reduced to reduce graphene oxide using ascorbic acid as a reducing agent, with the understanding that reduced graphene oxide is more reactive and conductive than graphene oxide.

Other research investigations have focused on silver sulfide quantum dots (Ag_2_S QDs) used in conjunction with graphitic carbon nitride nanosheets for the fluorescent detection of cerium (Ce) [44]. It is essential to highlight that Ag_2_S QDs are used in sensor fabrication because of the following properties: low toxicity, high quantum efficiency, low background fluorescence, and high photochemical stability [44]. Another report illustrated the use of lanthanum (La), which was assembled on graphitic carbon nitride nanosheets (CNNS) via a simple one-step hydrothermal method for fluorescent detection of Fe^3+^ [45]. The prepared La-CNNS was reported to possess high selectivity and sensitivity for fluorescence determination of Fe^3+^ with a wide linear range and low detection limit of 0.0232 µM [45]. In this regard, few reports have been documented in the literature on applying graphitic carbon nitride (g-C_3_N_4_) for sensor development. Table 1 gives a summary of recent reports using g-C_3_N_4_-based sensors for different analytes.

The reports obtained on the fabrication of various g-C_3_N_4_-based sensors to quantify different heavy metals revealed this smart nanomaterial’s efficacy. We strongly commend and recommend this material for various analytical applications. It is interesting to note that graphitic carbon nitride (g-C_3_N_4_) played fundamental roles in gas sensing. For this reason, the subject matter in the next paragraph is centered on the application of graphitic carbon nitride (g-C_3_N_4_) for gas sensors.

### 3.2. Fabrication of Gas Sensor Using Graphitic Carbon Nitride (g-C_3_N_4_)

Volatile organic compounds (VOC) or poisonous gases (PG) such as 2-butanone, ethanol, acetone, nitrogen (II) oxide, nitrogen (IV) oxide, and ammonia are incredibly harmful to human health and the environment. Human health is threatened by the combustion of organic fuels and leakages of toxic gases [46,47,48]. Methyl ethyl ketone (2-butanone) is a colorless liquid with a sharp, sweet odor, commonly used as a solvent in manufacturing processes to produce plastics, textiles, and paraffin wax, and in electronics. Still, its vapor can cause discomfort resulting in a coma or death [49]. According to several studies, nitric oxide (NO) gas is a hazardous gaseous pollutant produced through fuel combustion in automobiles and industrial activities [50].

However, due to technological advancement, VOC and PG concentrations have significantly increased over the past few years. Hence, it is crucial to develop cost-effective, green technology, and an efficient way of detecting these toxic gases in the environment. Different materials have played significant roles in detecting these notorious organic compounds and gases [51,52,53]. As mentioned earlier, graphitic carbon nitride (g-C_3_N_4_) has attracted much research interest due to its outstanding analytical features, including chemical inertness, physicochemical stability, suitable bandgap energy, ease of synthesis, ability to withstand harsh conditions, and high surface area for adsorption [54,55]. Graphitic carbon nitride (g-C_3_N_4_) performance for gas sensing can be improved by decorating, doping, and embedding various elements within its matrix [56,57].

For this reason, Cao et al. decorated a cocoon-like ZnO on graphitic carbon nitride (g-C_3_N_4_) via a simple hydrothermal technique for the detection of ethanol [58]. Polyethylene glycol (PEG) 400 was used as the surfactant during the synthesis, and the findings indicated that the cocoon-like ZnO were randomly distributed on the surface of the graphitic carbon nitride (g-C_3_N_4_) with a close interface between the nanomaterials [58]. The g-C_3_N_4_/ZnO nanocomposite-based sensor was operated under low operating temperatures at a wider linear concentration range of 100 to 2000 ppm [58]. Similarly, the p–n heterojunction (CuO-ZnO) was assembled on the surface of graphitic carbon nitride (g-C_3_N_4_) for ethanol sensing [59]. The morphologic results revealed that the flake-like CuO and ZnO nanoparticles were wrapped and intertwined by graphitic carbon nitride (g-C_3_N_4_) nanosheets. The ternary nanocomposite CuO-ZnO/g-C_3_N_4_ improved the sensing of ethanol, which was significantly higher (1.34 and 2.1 times) than that of CuO-ZnO and CuO [59]. The improvement in the sensing of ethanol by the ternary nanocomposite was attributed to the p–n heterojunction of CuO-ZnO and the brilliant substrate effect of graphitic carbon nitride (g-C_3_N_4_) nanosheet [59]. Notably, a heterogeneous composite of CuO/g-C_3_N_4_ was prepared by a facile ultrasound-assisted and calcination synthesis route [49]. This nanocomposite was used to construct a cataluminescent (CTL) sensor for 2-butanone [49]. The sensor was reported to demonstrate a rapid response and recovery time of 2 to 40 s, linear concentration ranges of 16.11–161.08 µg mL^−1^, and a detection limit of 11.06 µg mL^−1^ was obtained under the optimal experimental conditions [49].

In another report, palladium (Pd) nanoparticles were incorporated into the graphitic carbon nitride (g-C_3_N_4_). The nanocomposite was deposited on an interdigitated electrode (IDE) for the electrochemical detection of hydrogen gas using cyclic voltammetry (CV) [60]. The CV result showed significant hydrogen evolution and hydrogen oxidation reaction on the Pd/C_3_N_4_ nanocomposite. In addition, the current to voltage measurements confirmed that the Pd/C_3_N_4_ nanocomposite gave a better response towards hydrogen gas [60]. The author should have employed other electrochemical techniques such as square wave voltammetry or differential pulse voltammetry to validate the result obtained from the cyclic voltammetry technique.

Similarly, a palladium nanoparticle-graphitic carbon nitride (Pd/g-C_3_N_4_) composite was used for the fabrication of a hydrogen sensor; the graphitic carbon nitride (g-C_3_N_4_) was prepared by pyrolysis of melamine, while the palladium nanoparticles were uniformly dispersed on the surface of graphitic carbon nitride (g-C_3_N_4_) via polyol reduction technique [61]. The Pd/g-C_3_N_4_ composite displayed a high sensitivity for the detection of hydrogen gas at ambient temperature. The sensor’s sensitivity was 99.8%, with a response time of 88 s [61].

Furthermore, highly porous graphitic carbon nitride fibers were wrapped on gold nanoparticles to detect NO_2_ at ambient temperature [62]. The porous graphitic carbon nitride (g-C_3_N_4_) fibers were encapsulated on gold nanoparticles through a simple photochemical reduction process using HAuCl_4_ as the gold precursor, and sodium citrate played the roles of surface stabilizer and reducing agent. The gas sensor displayed good sensitivity/selectivity towards NO_2,_ with a detection limit of 60 ppb [62]. Additionally, density functional theory (DFT) calculations were used by Basharnavaz et al. to investigate the adsorption behavior of toxic NO gas on pristine graphitic carbon nitride (g-C_3_N_4_), Fe-, Ru- and Os-embedded g-C_3_N_4_ system [50]. The results revealed that the Os-embedded graphitic carbon nitride (g-C_3_N_4_) system exhibited high adsorption energy of −3.14 eV [50]. Therefore, NO gas’s adsorption energy was much higher on the Os-embedded graphitic carbon nitride (g-C_3_N_4_) system than other adsorbents [50].

Jang et al. developed a rapid ammonia gas sensor by in-situ polymerization of pyrrole on the surface of graphitic materials such as graphite, graphite oxide (GO), and reduced graphene oxide (rGO) [63]. The nanocomposite’s morphologic structure revealed interfacial interaction between polypyrrole (PPy) and graphitic carbon nitride (C_3_N_4_). The nanocomposite showed a significant response in detecting ammonia gas due to the effective electron charge transfer between PPy and ammonia gas [63]. The nanocomposite SEM images confirmed that the in-situ polymerization method assisted in blending/fusing the nanomediators.

In contrast, copolymerization interaction between guanidine hydrochloride and trimesic acid was used to synthesize Netty triazine-based graphitic carbon nitride (g-C_3_N_4_) [64]. The nanocomposite was employed for cataluminescent (CTL) detection of formic acid [64]. Notably, the control experiment showed that graphitic carbon nitride (g-C_3_N_4_) displayed excellent catalytic oxidation selectivity for formic acid. Therefore, the triazine-based graphitic carbon nitride (g-C_3_N_4_) was successfully used to detect formic acid [64].

### 3.3. Sensing of Organic Pollutants Using Graphitic Carbon Nitride (g-C_3_N_4_)

Over the past few decades, water contaminated by organic pollutants had become a significant concern to environmental scientists and industrial communities due to their applications in various fields such as pharmaceuticals, manufacture of dyes, explosives, anti-corrosion lubricants, medicine, and pigments, to name a few [65,66,67]. 4-nitrophenol (4-NP) is one of the most toxic organic compounds that affect different parts of the human body, including the mouth, skin, kidney, liver, respiratory, digestive, and central nervous system [65]. According to the United States Environmental Protection Agency (USEPA), 4-NP has been listed as a potential pollutant due to its severe toxicity to the environment and human health [68]. Most organic pollutants are potential mutagens, teratogens, and carcinogens, which affect plants, animals, and humans, even at a very low concentration [68]. Hence, it is crucial to develop a device to monitor these organic pollutants’ levels in water bodies and the environment.

**Figure 5:**
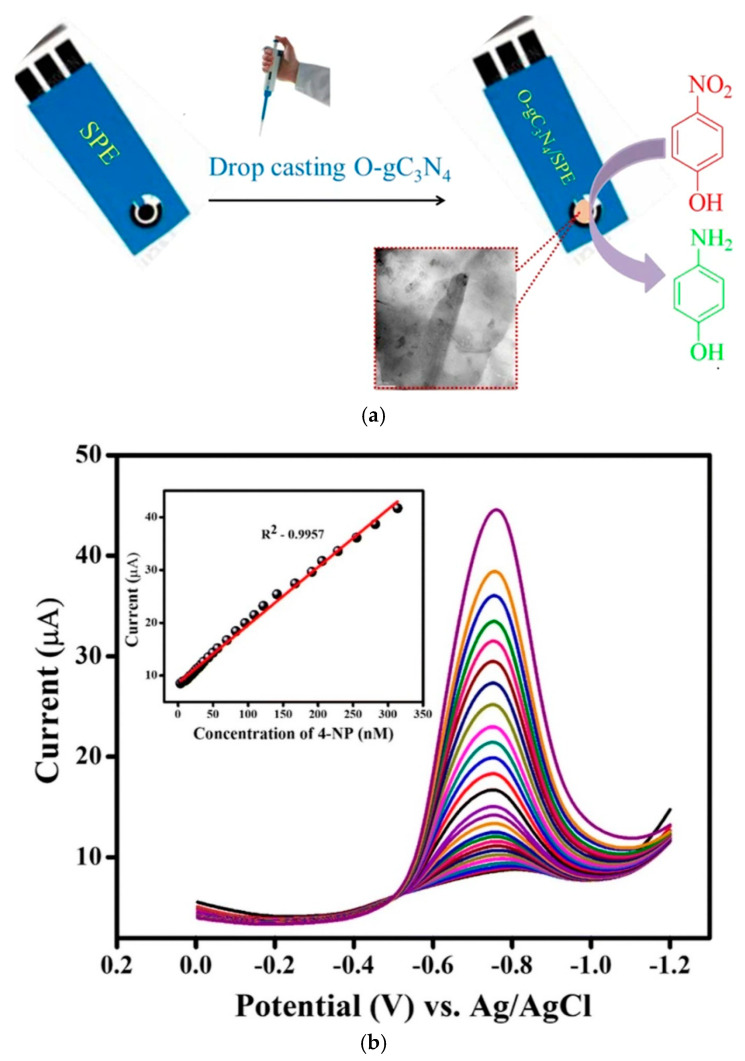
(**a**) Fabrication of 4-NP sensor and (**b**) DPV response of different concentrations of 4-NP (Copyright 2019 Springer; used with permission) [69].

For this reason, Ramalingam et al. developed an electrochemical sensor for the detection of 4-NP using oxidized graphitic carbon nitride (Ox–g-C_3_N_4_) [69]. The sensor was fabricated by drop-casting Ox–g-C_3_N_4_ on a screen-printed electrode (SPE). After that, cyclic voltammetry alongside differential pulse voltammetry (DPV) techniques was used to detect 4-NP. The Ox–g-C_3_N_4_/SPE was reported to possess excellent electrocatalytic activities towards 4-NP. The DPV response of the fabricated sensor showed a good linear range from 0.0033 to 0.313 µM, with a detection limit value of 0.075 µM [69]. It is important to note that graphitic carbon nitride (g-C_3_N_4_) was oxidized to incorporate some exciting functional groups, including carboxyl, hydroxyl, and ketone, which helped attract the analyte on the electrode surface. Thus, a lower detection limit was obtained. The construction of the 4-NP sensor and DPV results are highlighted in Figure 5a,b.

In another study, barium stannate was blended with graphitic carbon nitride (g-C_3_N_4_) by ultrasonic-assisted synthesis to form barium stannate–graphitic carbon nitride nanocomposite (BSO–gCN), and the resulting nanocomposite was used for the electrochemical detection of 4-NP via electro-oxidation [68]. The prepared BSO–gCN was drop-cast on a pre-treated glassy carbon electrode for the electrochemical quantification of 4-NP using linear sweep voltammetry (LSV) technique. The following thermodynamic parameters were reported: charge transfer coefficient (α) of 0.5, rate constant (k_s_) of 1.16 s^−1^ and detection limit and sensitivity of 1 µM and 0.81 µA µM^−1^ cm^−2^ were obtained [68]. It is interesting to note that the sensor was selective and sensitive for detecting 4-NP in the presence of a 100-fold excess ion (Ca^+^, K^+^, Na^+^, I^−^, Cl^−^, NH_4_^+^, and SO_4_^2−^) in the same solutions with 4-NP [68]. The chemistry and the interaction of graphitic carbon nitride (g-C_3_N_4_) and barium stannate should be investigated. In addition, other electrochemical techniques must be used to validate the result obtained by the linear sweep voltammetry technique.

Zhao et al. employed poly(diallyl dimethylammonium chloride) and graphitic carbon nitride-ionic liquid to modify a carbon paste electrode for the detection of tetrabromobisphenol A (TBBPA) [70]. The N-butyl pyridinium hexafluorophosphate (NBH), an ionic liquid, was reported to promote the charge transfer of the graphitic carbon nitride (g-C_3_N_4_). The TBBPA was detected using differential pulse voltammetry (DPV) in the range of 1 nM to 30 nM and 30 to 500 nM with a detection limit of 0.4 nM [70]. The interaction between the positive charge of PDDA and the negatively charged hydroxyl functional group on the TBBPA enhanced the sensor’s response signal. It is important to note that TBBPA is an endocrine disruptor organic pollutant and increases the risk of tumors in the body [70]. The electrode reaction mechanisms that occurred during the detection of TBBPA are presented in Equations (3) and (4).

HO − TBBPA–OH- e^−^ → HO − TBBPA=O (3)

HO − TBBPA=O- e^−^ → O=TBBPA=O (4)

In another report, a photoelectrochemical sensor for bisphenol A (BPA) was constructed using Cu/graphitic carbon nitride nanocomposites [71]. The Cu/g-C_3_N_4_ composite was prepared via a solvothermal method in copper-based ionic liquid [71]. The BPA photoelectrochemical sensor displayed excellent stability and satisfactory anti-interference properties with a detection limit of 0.012 µmol/L [71]. The copper nanomaterials helped suppress the recombination of photogenerated electron–hole pairs during BPA detection.

In another study, carboxylated graphitic carbon nitride (C–g-C_3_N_4_) and NH_2_-aptamer were used to develop a novel electrochemiluminescence (ECL) sensing scaffold for excellent selective and sensitive detection of bisphenol A (BPA) [72]. The NH_2_-aptamer was covalently bonded to the carboxylated graphitic carbon nitride (g-C_3_N_4_) via covalent attachment. In the presence of bisphenol A, the ECL signal decreases due to electrochemiluminescence energy transfer from the excited state of the nanocomposite to the oxidation product of bisphenol A [72]. The aptamers employed in this study are single-stranded oligonucleotide molecules selected from combinatorial libraries that have the ability and capacity to bind with target and specific analytes [73]. The significant analytical properties of the aptamers are high affinity, selectivity, and specificity [73]. The electrostatic attraction between the negatively charged carboxylated graphitic carbon nitride (g-C_3_N_4_) and the positively charged NH_2_-aptamer helped obtain a lower detection limit.

One-step electrochemical synthesis of graphitic carbon nitride/graphene hybrid was used to develop an ECL sensor for the detection of pentachlorophenol (PCP), a group 2B environmental carcinogenic organic pollutant [74]. In this work, graphene did not only assist in the immobilization of the graphitic carbon nitride (g-C_3_N_4_) but also helped enhance the ECL signal by approximately 4.7 times. The sensor was used to detect PCP in a real water sample with a satisfactory recovery rate [74].

Recent studies have shown that graphitic carbon nitride (g-C_3_N_4_) played fundamental roles in the simultaneous detection of organic pollutants. For instance, a one-step thermal polymerization synthesis route was used for the synthesis of gold nanoparticles/g-C_3_N_4,_ which was further used for the simultaneous determination of hydroquinone (HQ) and catechol (CC) [75]. The sensor was assembled by modifying a glassy carbon electrode with the nanocomposite and used for the simultaneous detection of HQ and CC in spiked water samples [75]. The remarkable properties of the sensor were attributed to the synergy between the microstructure (porosity and heterojunction) alongside with the π-interaction between the phenolic isomers and graphitic carbon nitride (g-C_3_N_4_) [75]. In our view, Nafion was supposed to be used as the stabilizing agent. However, the author electrodeposited gold nanoparticle as a smart modifying route to stabilize the modifier on the electrode surface.

Graphitic carbon nitride-based nanohybrid materials can be used as photocatalyst and for the fabrication of an electrochemical sensor. For instance, Mohammad et al. employed zinc oxide-graphitic carbon nitride (ZnO–CN) for the detection of multiple nitroaromatics, including 4-nitrotoluene (4-NT), 2,4-dinitrotoluene (2,4 DNT) and 2,4,6-trinitrophenol (2,4,6-TNP) [76]. The nanocomposite was used for the photocatalytic degradation of Chicago sky blue (CSB), Congo red (CR), and methylene blue (MB) [76]. The ZnO–CN nanohybrid was reported to possess a high surface area. The heterojunction formed between the interfaces of ZnO and g-C_3_N_4_ helps to facilitate electron transfer and the separation of photo-induced electron-hole pairs [76]. Table 2 shows recent reports on the application of graphitic carbon nitride (g-C_3_N_4_) to detect organic pollutants.

It is essential to highlight that the disparity in the detection limits reported in Table 2 is dependent on the following reasons: (1) the techniques used for the analyte detection, (2) the nanomaterials used to modify the conducting substrate, (3) the conductivity of the nanomaterials incorporated into the matrix of graphitic carbon nitride (g-C_3_N_4_) (4) the conducting substrate used for the detection of the analyte. For instance, a detection limit of 0.075 nM was obtained when OX-g-C_3_N_4_ was used to detect 4-NP, which is far lower compared to a detection limit of 1000 nM obtained using BSO–gCN. The reason for this apparent disparity can be attributed to the technique used, with the understanding that DPV is more sensitive than CV, while SWV is more sensitive than DPV. The oxidation of the graphitic carbon nitride (g-C_3_N_4_) helped attract the analyte on the conducting substrate.

### 3.4. Detection of Pharmaceuticals Using Carbon Nitride (g-C_3_N_4_)

At the time of writing this review, a respiratory illness caused by a novel coronavirus (COVID-19) has infected tens of thousands of people in many parts of the world. It is sending scores of people to their early graves. Pharmaceutical companies and research centers worldwide are working hard to develop drug treatments to help those who fall ill. Pharmaceuticals play a vital role in the treatment of diseases in animals and humans. The pharmaceutical industry’s new cures and treatment options are crucial to combat the myriad of diseases affecting humans, such as human immunodeficiency virus (HIV), Ebola virus, hypertension, strokes, tuberculosis, cancer, measles, diabetes, and coronavirus. However, pharmaceutical residues are released into the environment through various routes during their manufacture and the disposal of unused pharmaceuticals. When pharmaceuticals are ingested or taken orally by animals and humans, they are not entirely metabolized and are discharged via feces or urine to the environment [77]. The presence of pharmaceutical residues was first reported in wastewaters and surface waters in Europe and the United States in the 1960s [77]. However, a tremendous increase in pharmaceuticals in wastewaters, groundwater, and surface water has been observed since then [78]. Hospitals and pharmaceutical industries make a significant contribution to the presence of pharmaceutical waste in water bodies. In addition, household waste has been reported to contain pharmaceuticals due to improper disposal of expired or unused drugs and excretion of unmetabolized medicines [78,79].

Different pharmaceuticals have been detected in various water bodies, primarily antibiotics and pain relief tablets such as paracetamol, ciprofloxacin, sulfamethoxazole, and sulfadimethoxine were reportedly found in concentrations as high as the milligram per liter range in water [77]. For instance, ciprofloxacin is used for treating bacterial infections in both humans and animals; it has been detected at a concentration of 50 mg/L in effluents of factories producing ciprofloxacin [77]. Consumption of water containing high levels of ciprofloxacin can result in nausea, diarrhea, vomiting, and acute renal failure [77]. Therefore, it is crucial to monitor the concentration of pharmaceuticals in various water bodies quantitatively.

**Figure 6:**
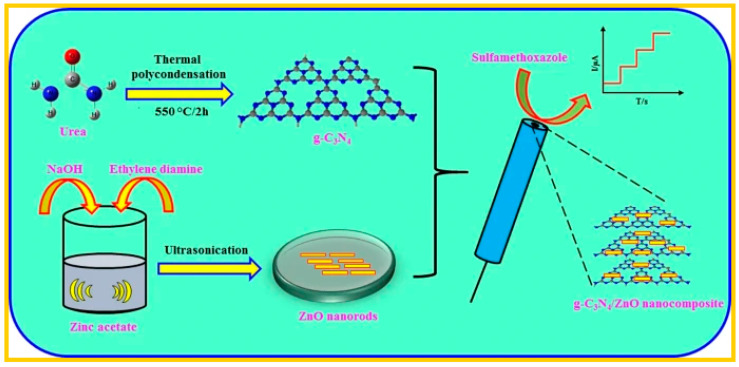
Synthesis and the application of g-C_3_N_4_/ZnO for the detection of sulfamethoxazole. (Copyright Springer 2019, with permission [80].

For this reason, a voltammetric sensor was designed for the detection of sulfamethoxazole (SMZ) by modifying a glassy carbon electrode (GCE) with a nanocomposite prepared from graphitic carbon nitride (g-C_3_N_4_) and zinc oxide nanostructure (g-C_3_N_4_/ZnO) [80]. The nanorod-like ZnO nanostructure was sonochemically prepared, and the g-C_3_N_4_/ZnO nanocomposite was prepared by mixing g-C_3_N_4_ and ZnO nanostructure followed by ultrasonication. After this, the nanocomposite was drop-cast on the GCE to determine SMZ in spiked human blood serum samples, and the result obtained was satisfactory [80]. Figure 6 showed the scheme for the synthesis and application of g-C_3_N_4_/ZnO nanocomposite for the detection of sulfamethoxazole.

Yan et al. reported the photoelectrochemical (PEC) detection of ciprofloxacin (CP) using nitrogen-deficient graphitic carbon nitride (ND-g-CN) [81]. The PEC sensor was constructed by drop-casting ND-g-CN on indium tin oxide, and the sensor was responsive to a wide CP concentration range of 60 to 19,090 ng/L with a detection limit of 20 ng/L [81]. It is essential to highlight that the nitrogen vacancies act as a charge trap to hinder charge transfer separation effectively and widen the adsorption edge and decrease the bandgap of the ND-g-CN. Thus, resulting in increasing light-harvesting and enhancing the performance of the photoelectrochemical sensor [81].

Dang et al. designed a novel photoelectrochemical (PEC) aptasensor for sulfadimethoxine using graphitic carbon nitride (g-C_3_N_4_) quantum dots (QDs) and reduced graphene oxide (rGO) to modify fluorine-doped SnO_2_ (FTO) electrodes [82]. The aptamer was immobilized on the electrode through π-stacking interaction. The graphitic carbon nitride (g-C_3_N_4_) quantum dots were reported to possess the following properties: (1) high emission quantum yield, (2) better charge separation capability, and (3) effective optical absorption [82]. The rGO contributed to the electron transfer kinetics of the PEC sensor. Cumulatively, these features aided in improving the PEC performance [82]. In another report, a fluorescent quenching sensor was exploited for the detection of chlortetracycline (CTC) using a combination of graphitic carbon nitride (g-C_3_N_4_) with molecular imprinted silica [83]. The sensor’s fluorescence quenching property was due to the chemistry between the graphitic carbon nitride (g-C_3_N_4_) and the benzene ring of CTC via π-π electron donor-acceptor interaction and electrostatic force [83].

Moreover, a novel electrochemical sensor was developed using PEDOT/graphitic carbon nitride (g-C_3_N_4_/PEDOT-MESH) composites for the detection of paracetamol (PAR), which is widely used in most homes due to its significant roles in antipyretic and analgesic activities [84]. The sensor was fabricated by dispersing 1 mg of the nanocomposite into equal volumes (5 mL) of isopropanol and distilled water to form a homogeneous suspension. After that, Nafion film was used to drop-cast the suspension on a glassy carbon electrode’s surface to improve the stability and prevent the nanocomposite’s leaching in the solution [84]. It is of utmost importance to highlight that the synergistic effects of PEDOT-MeSH and graphitic carbon nitride (g-C_3_N_4_) enhanced the electron transfer and conductivity of the modified electrode as well as improved the electrochemical oxidation of paracetamol [84]. The sensor displayed a wide linear response range of 2–1680 μM, and a detection limit of 1 μM was obtained [84]. The Nafion film was used to stabilize the nanomaterials on the conducting substrate. However, the PEDOT would have been electrodeposited on the GCE after drop-drying the graphitic carbon nitride (g-C_3_N_4_) on the electrode surface; this would have helped to reduce the cost of the sensor and condense the preparation step.

Despite the impressive performance of graphitic carbon nitride (g-C_3_N_4_) for the quantification of pharmaceuticals in water, only scant reports have been documented on its application for sensing pharmaceuticals in water; we strongly recommend its usage for the quantification of pharmaceuticals in water on the premise of the remarkable results reported in the literature. Table 3 shows the reports for the detection of pharmaceuticals in water.

### 3.5. Sensing of Biologic Molecules Using Graphitic Carbon Nitride (g-C_3_N_4_)

#### 3.5.1. Sensing of Glucose

Several methods have been used to prevent, reduce, and treat various life-threatening diseases such as diabetes, cancer, atherosclerosis, cardiomyopathy, stroke, and heart attack [85,86]. Early diagnosis, coupled with efficient and effective monitoring of glucose levels in the body, is vital to keep diabetes under control [87,88]. Currently, the number of patients suffering from various diseases has increased exponentially. At the time of compiling this review, an outbreak of respiratory illness caused by a novel coronavirus (COVID-19) in many countries around the world has resulted in large numbers of people being hospitalized, and many deaths have been reported; the World Health Organization (WHO) on 11 March 2020 declared the COVID-19 outbreak a pandemic. There is, therefore, high demand for early clinical diagnosis and treatment of various forms of diseases. For this reason, Ngo et al. reported a novel photoluminescence glucose sensor-based on aminoboronic acid-functionalized graphitic carbon nitride quantum dots (g-CNQDs/3APBA) [89]. The sensor’s selectivity was interrogated using various interfering species, including ascorbic acid, sorbitol, L-cystine, uric acid, and glycine. The g-CNQDs/3APBA displayed excellent selectivity towards glucose over other biologic molecules due to the specific reaction between the 1,2 diols of glucose and the APB’s boronic acid. The nanocomposite was recommended as a sensing material for biomedical and clinical applications [89]. Similarly, Ngo and co-workers reported highly biocompatible phenylboronic acid-functionalized graphitic carbon nitride quantum dots to detect glucose [90]. Here, the graphitic carbon nitride (g-C_3_N_4_) was used as a fluorescent nanomaterial, while the phenylboronic acid acted as the quencher and the receptor for the glucose sensor [90]. A detection limit of 16 nM was obtained, which was an improved electrochemical performance compared to the glucose sensor based on aminoboronic acid-functionalized graphitic carbon nitride quantum dots (g-CNQDs/3APBA) with a detection limit of 40 nM [89]. The constructed biosensor’s selectivity was evaluated on ascorbic acid, sorbitol, dopamine, L-cystine, and other saccharides present in real blood. All the biologic molecules showed no significant response towards g-CNDs/PBA; this is due to the specific reaction between glucose and PBA by the presence of a cis-diol pair at the 1,2-and 5,6- positions in glucose [90].

In another report, a unique nonenzymatic glucose sensor was designed on the premise of depositing nanolayered Co(OH)_2_ on polymeric graphitic carbon nitride (Co(OH)_2_-g-C_3_N_4_) via chemical bath deposition route [91]. The 2D nanocomposite was used to modify a carbon paste electrode for the electrochemical oxidation of glucose using electrochemical impedance spectroscopy (EIS) and amperometry techniques. The concentration ranges of 25 μM–420 mM and 6.6–9800 μM were used for the EIS and amperometry methods. The sensor was used for the quantification of glucose in blood serum samples [91]. The selectivity of the biosensor was studied using chronoamperometry. This experiment was carried out by successive addition of 0.001 mM dopamine, uric acid, glutathione, urea, ascorbic acid, and glucose to 0.2 M KOH solution using an applied potential of 0.6 V. The results showed that the presence of these biomolecules did not cause any significant reduction in the peak current signal of glucose [91].

In addition, graphitic carbon nitride (g-C_3_N_4_) nanosheets were decorated with iron oxide and copper nanostructures derived from Cu_3_[Fe(CN)_6_]_2_ for the development of nonenzymatic glucose detection [92]. The nanocomposite (g-C_3_N_4_/Fe_2_O_3_–Cu) was prepared using Prussian blue Cu_3_ [Fe(CN)_6_]_2_ and melamine as precursors. The sensor was reported to exhibit excellent stability owing to the presence of Cu-nanostructure and thin-layered graphitic carbon nitride (g-C_3_N_4_) nanosheets. The results showed that the nanocomposite (g-C_3_N_4_/Fe_2_O_3_–Cu) possesses impressive anti-interference activity, long-term stability, and excellent reproducibility features [92]. The sensor displayed unique electrochemical properties owing to the bimetallic synergy of Cu and Fe in the nanocomposite.

In another study, g-C_3_N_4_/Fe_3_O_4_ magnetic nanocomposites were prepared by chemical co-precipitation of graphitic carbon nitride and magnetite (Fe_3_O_4_). The nanocomposite was used in the determination of glucose and hydrogen peroxide (H_2_O_2_) [93]. The g-C_3_N_4_/Fe_3_O_4_ magnetic nanocomposites showed better electrochemical activity when compared to g-C_3_N_4_ or Fe_3_O_4_ NPs. Interestingly, the sensor was reported to catalyze the oxidation of different peroxidase substrates, including 3,3,5,5-tetramethylbenzidine (TMB), o-phenylenediamine (OPD), and 2,2-azino-bis(3-ethylbenzothiazoline-6-sulfonic acid) diammonium salt (ABTS) by H_2_O_2_ to produce different colors, including blue, orange, and green. The sensor was used to determine glucose in spiked human serum, and detection limits of 0.25 µM and 0.3 µM were obtained for glucose and hydrogen peroxide, respectively [93]. The sensor’s selectivity was interrogated using common co-existing substances, including NaCl, KCl, CaCl_2_, cysteine, dopamine, ascorbic acid, Cr_2_O_7_^2−^ and ClO^−^. The result obtained confirmed that these substances did not influence the determination of H_2_O_2_ [93].

**Figure 7:**
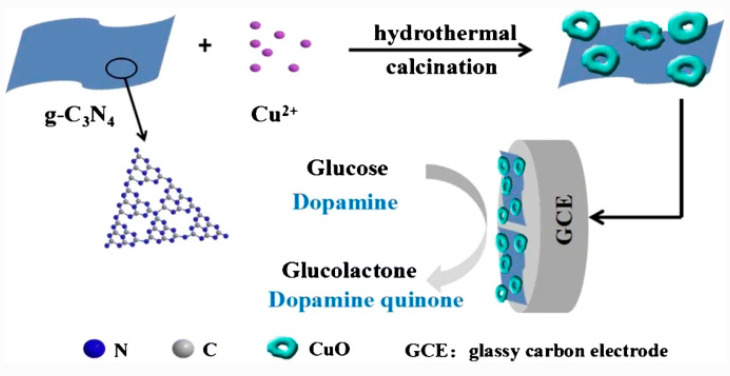
Synthesis and the application of graphitic carbon nitride for the simultaneous detection of glucose and dopamine [94].

Huang et al. successfully prepared three kinds of carbonaceous nanocomposites (CuO/g-C_3_N_4_), (CuO/porous carbon) and (CuO/carbon sphere) by hydrothermal synthesis [94]. The nanocomposites were used in modifying a glassy carbon electrode (GCE) for the simultaneous detection of glucose and dopamine. The results showed that the GCE modified with CuO/g-C_3_N_4_ displayed the highest electrocatalytic activity towards glucose and dopamine due to the synergistic interactions between CuO and g-C_3_N_4,_ which helped in enhancing the conductivity of the sensor [94]. The prepared sensor’s selectivity was investigated by adding 0.1 mM interferent species (dopamine, ascorbic acid, fructose, uric acid, and AAP) into a solution containing 1.0 mM glucose prepared in 0.1 M NaOH. It was reported that the current changes caused by the interferent species are negligible. The proposed scheme for sensor fabrication is depicted in Figure 7.

#### 3.5.2. Sensing of Dopamine and Other Biomolecules

Dopamine (DA) is a naturally occurring biological molecule or neurotransmitter present in the central nervous system [95]. It plays a crucial role in motivation, cognition, and endocrine regulation [96]. It is important to note that there is a close relationship between concentrations of DA and several diseases, including Parkinson’s disease and schizophrenia [97]. In biological systems, DA’s average concentration in the body is 10^−8^ and 10^−6^ M [98]. It was reported that the abnormal production of DA in the brain is associated with neurological disorders, including Huntington‘s disease and attention-deficit hyperactivity [98]. Therefore, monitoring the concentration of DA in the body has been prioritized in biomedical and analytical fields.

For this reason, Jiang et al. designed a simple electrochemical biosensor for dopamine detection by the immobilization of graphitic carbon nitride (g-C_3_N_4_) on a glassy carbon electrode [98]. Here, the biosensor was prepared by casting graphitic carbon nitride (g-C_3_N_4_) Nafion solution onto the surface of a glassy carbon electrode and allowing it to dry at ambient temperature. The stability and reproducibility of the biosensor were evaluated in phosphate-buffered saline (PBS) solution; the decrease in the sensor’s electrochemical activity was 10% after 15 days, this confirmed that the fabricated biosensors are promising tools for detecting and monitoring DA concentrations [99]. The biosensor’s stability was attributed to the Nafion solution used to prevent the leaching of the graphitic carbon nitride (g-C_3_N_4_) in the analyte solution. Hence, we recommend that the Nafion solution should be added to the preparation step during sensor development.

In addition, calcium stannate was incorporated into graphitic carbon nitride (g-C_3_N_4_) to develop an electrochemical sensor to quantify dopamine [100]. The following thermodynamic parameters were obtained for the dopamine sensor using linear sweep voltammetry: charge transfer coefficient (α) = 0.58, rate constant for electron transfer (K_s_ = 0.95 s^−1^) and linear range = 0.1 to 1 mM. The differential pulse voltammetry (DPV) was used to detect dopamine with a detection limit and sensitivity of 29 μM and 0.0057 μA mM^−1^ cm^−2^ [100]. It is important to highlight that different organic and inorganic interference species (Na_2_SO_4_, NaNO_3_, NaNO_2_, NaCl, urea, uric, and glucose) were present in detecting dopamine and were found not to interfere in the detection of DA [100].

In another report, gold nanoparticle was decorated on a water-soluble anionic dihydroxylatopillar [5] arene (2HP5)-functionalized graphitic carbon nitride (g-C_3_N_4_) for the electrochemical detection of dopamine [101]. The nanocomposite was synthesis by π-interaction between water-soluble-2HP5 and graphitic carbon nitride (g-C_3_N_4_). The nanocomposite helps to host the DA biomolecules on the electrode surface.

Hydrogen peroxide (H_2_O_2_) is a chemical compound that is increasingly receiving attention because of its application in the food and medical field. Furthermore, hydrogen peroxide (H_2_O_2_) is an essential intermediate in the food and chemical industries that play fundamental biological and life processes [102,103,104,105,106]. Ernstgård et al. investigated the effects of inhaled hydrogen peroxide vapors in humans; findings suggested that hydrogen peroxide is slightly irritating at 2.2 ppm, but not at 0.5 ppm [103]. Hydrogen peroxide is a byproduct of cellular metabolism that causes oxidative stress at higher concentrations [105].

The reports on the toxicological study on hydrogen peroxide revealed that its exposure could cause oxidative stress, irritation of the eyes, respiratory inflammation, Alzheimer’s, diabetes, physiological and neurodegenerative disorders [106]. Therefore, the detection and determination of hydrogen peroxide are crucial in clinical and analytical fields.

A paper-based analytical device (PAD) was used to detect hydrogen peroxide in milk samples by Lima et al. [102]. The method is based on the chemical reaction between hydrogen peroxide and guaiacol. This reaction is catalyzed by peroxidase to produce a red substance that is quantified by digital imaging. The device offered advantages such as low cost, simplicity, and portability. This platform was successfully used to quantify hydrogen peroxide with a recovery rate of 92.2% and 109% [102].

Ahmed et al. fabricated sponge-like graphitic carbon nitride (g-C_3_N_4_) and silver oxide nanocomposites for the fluorometric quantification of H_2_O_2_ [105]. The silver oxide (Ag_2_O) was reported to help improve the nanocomposite’s electrical properties by reducing the recombination of photogenerated holes and electron pairs, which resulted in enhancing the biosensor’s sensitivity. The developed fluorescence biosensor was highly sensitive and selective towards the detection of H_2_O_2_ with a linear range of 30–300 nM and a detection limit of 22 nM [105].

In another report, hierarchically structured ZnO was decorated on graphitic carbon nitride (g-C_3_N_4_) for the fabrication of nonenzymatic photoelectrochemical (PEC) detection of H_2_O_2_ [106]. The PEC sensor measures reduction in the photocurrent response by the H_2_O_2,_ which acts as a scavenger to the photoinduced electrons produced by the nanocomposites (ZnO/g-C_3_N_4_). The graphitic carbon nitride (g-C_3_N_4_) extends the bandwidth of light absorption of the zinc oxide into the visible region and helps separate the photoinduced carriers; this contributed significantly to the enhancement in the photocurrent response and improvement in the sensitivity of the PEC sensor [106]. The graphitic carbon nitride (g-C_3_N_4_) formed a heterojunction with the ZnO, which helped improve the PEC sensor’s sensitivity by retarding the recombination of the photogenerated electron-hole pair.

Notably, graphitic carbon nitride (g-C_3_N_4_) nanosheets have been prepared via direct pyrolysis of melamine, followed by protonation and ultrasonication. The graphitic carbon nitride (g-C_3_N_4_) nanosheets were used for nonenzymatic electrochemical detection of hydrogen peroxide and paracetamol. The graphitic carbon nitride (g-C_3_N_4_) nanosheet was reported to exhibit a large specific surface area and promote the ionic activity of the sensor [107]. The density functional theory (DFT) was used to interrogate the electrical properties of the graphitic carbon nitride (g-C_3_N_4_) nanosheets; the result confirmed that the protonation enhanced the conductivity of the sensor. The advantage of the fabricated sensor includes high sensitivity, superior electrocatalytic properties, fast response time, and excellent stability [107]. It is essential to highlight that different morphology of graphitic carbon nitride (g-C_3_N_4_) exhibit various analytical features.

Due to the fascinating and remarkable electrocatalytic properties, graphitic carbon nitride (g-C_3_N_4_) played vital roles in developing a sensor for cancer biomarkers. For example, graphitic carbon nitride (g-C_3_N_4_) was used in the development of a split protocol for the photoelectrochemical (PEC) immunoassay of alpha-fetoprotein (AFP)-a cancer biomarker [108]. The PEC immunoassay was used to detect AFP in serum samples [108]. Similarly, cysteine-assisted g-C_3_N_4_–BiOCl and CuO nanoparticles were immobilized on the surface of an ITO electrode in the photoelectrochemical immunoassay for carcinoembryonic antigen (CEA)- a cancer biomarker [109]. The heterojunction formed between g-C_3_N_4_ and BiOCl assisted in increasing the photocurrent response of the PEC immunoassay [109]. We have developed various electrochemical immunosensors for the detection of AFP [110,111,112]. In 2019, we developed an exfoliated graphite-based electrochemical immunosensor on a dendrimer/carbon nanodot platform to detect the carcinoembryonic antigen (CEA) cancer biomarker [113].

**Figure 8:**
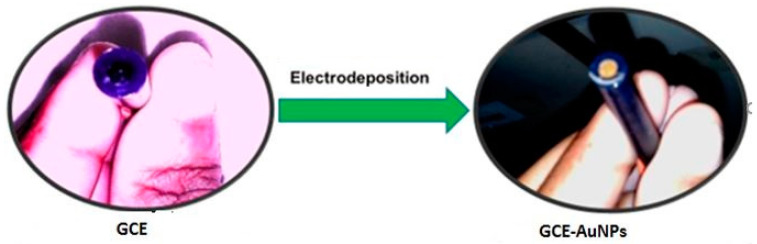
Electrodeposition of gold nanoparticles (AuNPs) on GCE [32,33,34].

Luo et al. reported a PEC sensor for the detection of human epidermal growth factor receptor 2 (HER2)-breast cancer biomarker by the electrodeposition of gold nanoparticles on the surface of hexagonal carbon nitride tubes (HCNT) [114]. The HCNT was prepared by a facile hydrothermal method with low electron-hole recombination and large specific surface area. After this, gold nanoparticles were deposited on the surface of the HCNT, which helped enhance the photocurrent response of the HCNT [114]. We reported the electrodeposition of gold nanoparticles (AuNPs) on conducting substrate by cycling a potential from −400 to 1100 mV for ten cycles at a scan rate of 50 m/s [32,33,34]. Figure 8 reveals how we electrodeposited AuNPs on a glassy carbon electrode (GCE).

Similarly, gold nanoparticles (AuNPs)—in synergy with graphitic carbon nitride quantum dots (g-CNQDs)—were used in the construction of an electrochemiluminescence (ECL) sensor for ultrasensitive detection of DNA [115]. The g-CNQDs were used as the energy transmitter, and AuNPs played the role of a quencher in the fabrication of the ECL-resonance energy transfer-based DNA biosensor [115]. The biosensor was sensitive within the linear range of 0.02 fM to 0.1 pm, and a detection limit of 0.01 fM (3σ) was obtained. More importantly, the authors reported that few reports had been documented in the literature on the application of g-CNQDs for ECL fields. We strongly recommend graphitic carbon nitride (g-C_3_N_4_) to scientific and industrial communities for this application.

In addition, graphitic carbon nitride (g-C_3_N_4_) nanosheets have been assembled on a porous gold electrode and used to develop an ultrasensitive electrochemiluminescence immunosensor for DNA [116]. Here, the gold electrode provided numerous binding sites for the target biomolecules and assisted in incorporating many DNA labels. The graphitic carbon nitride (g-C_3_N_4_) nanosheets were used to create a super-sandwich-type assembly on the gold electrode via DNA hybridization process. The ECL immunosensor was reported to display impressive performance in a concentration range from 0.01 fg/mL to 1 μg/mL with a remarkable detection limit of 0.001 fg/mL.

Apart from these aforementioned biomolecules, graphitic carbon nitride (g-C_3_N_4_) has also played fundamental roles in detecting uric acid (UA). It is an effective antioxidant that eliminates single oxygen and free radicals in human blood [117]. It is known that a slight change in the concentration of UA levels in human fluids may lead to hyperuricemia, podagra, and cancer [117]. For this reason, a porous graphitic carbon nitride (g-C_3_N_4_) was used to design a fluorescent probe for the determination of trace uric acid in human serum and human plasma [118]. The authors reported that the results obtained were consonant with UA standard solutions [118]. Among the most recent developments in the utilization of graphitic carbon nitride-based materials for biomolecule sensing, the innovation that stands out was the development of an electrochemical sensor for the simultaneous detection of ascorbic acid (AA), dopamine (DP), and uric acid (UA) [119]. The sensor was constructed by immobilizing porous graphitic carbon nitride (g-C_3_N_4_) nanosheets (PCN) wrapped with graphene oxide (GO) on a glassy carbon electrode to monitor AA, DP, and UA in spiked serum samples [119]. The PCN was reported to increase the specific surface area, increase the active sites, and enhanced the catalytic properties of the electrochemical sensor. At the same time, the GO improved the conductivity of the PCN and increased the electrocatalytic activity of the nanocomposite [119]. Due to the synergistic properties of both nanocomposites, the fabricated electrochemical sensor possesses a high specific surface area, hierarchical pore structure, and remarkable signal response to the biologic molecules (AA, DP, and UR). The application of g-C_3_N_4_@GO nanocomposite for the simultaneous detection of AA, DP, and UR was possible because of well-separated peaks at oxidation potentials of 0.15, 0.34, and 0.46 V, respectively [119]. The electrochemical sensor was applied in tracking the biomolecules in spiked human serum with a recovery rate of 95.1 to 105.5%. Table 4 presents the application of graphitic carbon nitride for the detection of biologic molecules.

## 4. Conclusions and Recommendation

This review summarizes the recent advances in graphitic carbon nitride-based sensors for various applications, including heavy metals, gas sensing, organic pollutants, pharmaceuticals, and biomolecules. The impressive performance of graphitic carbon nitride (g-C_3_N_4_) is primarily based on its surface state (doping, defects, and functional groups) and structures (thickness, porosity, and morphology). The use of graphitic carbon nitride (g-C_3_N_4_) in the development of various sensors is based on its analytical properties, including high surface area, low cost, environmentally friendly non-toxic, and excellent biocompatibility. Despite the impressive results reported on using graphitic carbon nitride (g-C_3_N_4_) as a smart and intelligent nanomaterial for electrochemical applications, only a few reports are documented in the literature for its usage in the fabrication of sensors for heavy metals, gas sensing, organic pollutants, pharmaceuticals, and biomolecules. This review urges scientific and industrial communities to explore this underused graphitic carbon nitride (g-C_3_N_4_) to develop various sensors.

However, we recommend the following: (i) The starting material or the precursors used for the synthesis of graphitic carbon nitride should be environmentally friendly and affordable; (ii) The electrodeposition modification route should be exploited in the deposition of graphitic carbon nitride (g-C_3_N_4_) on the electrode surface, which is far better than the drop-drying and the drop-coating method reported in the literature; (iii) It is essential to use at least two electrochemical techniques to validate the results obtained when graphitic carbon nitride (g-C_3_N_4_) is used as a modifier or other modifiers doing electrochemical analysis; (iv) It is essential to understand the chemistry and interaction that takes place when other nanomaterials are incorporated into the matrix of graphitic carbon nitride (g-C_3_N_4_); (v) Finally, it is necessary to add stabilizing agents such as Nafion solution as one of the fabrication steps to prevent the leaching of the nanomaterial inside the analyte solution during electrochemical analysis.

## Figures and Tables

**Table 1:** Recent reports on g-C_3_N_4_-based sensors.

Analyte	Method	Linear Range (nM)	LOD (nM)	Materials	Ref
Hg	Chemosensor	0.25–10	0.08	g-CNQDs	[30]
Hg	Colorimetry	100–500	0.275	Au@S-C_3_N_4_	[31]
Hg	Fluorescence	1–1000	0.3	GCNNS	[35]
Hg	Fluorescence	10^3^–3 × 10^4^	140	g-C_3_N_4_	[36]
Hg	Fluorescence	100–8 × 10^3^	94	g-C_3_N_4_	[37]
Hg	Fluorescence	0.5–100	0.17	g-C_3_N_4_	[38]
Hg	SWASV	0.06–25	0.018	IP-GCNT	[39]
Ag	Electrochemical	0.001–100	0.0009	GCNNS	[40]
Cd	SWASV	50–700	3.9	GCNNS	[41]
Cu	PEC	1–100	0.38	g-C_3_N_4_	[42]
Cu	ECL	10^−8^–10^−4^	10^−8^	GCN-GO	[43]
Ce	Fluorescence	0.0005–0.032	6.4 × 10^−5^	Ag_2_S QD-g-C_3_N_4_	[44]
Fe	ECL	100–20,000	23.2	La-CNNS	[45]

g-CNQDs—graphitic carbon nitride quantum dots; Au@S-C_3_N_4-_—gold@sulfur-doped graphitic carbon nitride; GCNNS—graphitic carbon nitride nanosheets; GCN-ssDNA—graphitic carbon nitride single-stranded DNA; IP-GCNT—imprinted graphitic carbon nitride; 3DBC-GCN—three-dimension branched crystalline graphitic carbon nitride; GCN-GO—graphitic carbon nitride-graphene oxide; Ag_2_S-GCNNS—silver sulfide quantum dots–graphitic carbon nitride nanosheets; La-GCNNS-lanthanum graphitic carbon nitride nanosheets.

**Table 2:** Recent reports on g-C_3_N_4_-based sensors for organic pollutants.

Analyte	Method	Linear Range (nM)	LOD (nM)	Materials	Ref
4-NP	LSV	1600–50,000	1 000	BSO–gCN	[68]
4-NP	DPV	3.3–313	0.075	OX-g-C_3_N_4_	[69]
TBBPA	DPV	1–30	0.4	PDDC–g-C_3_N_4_	[70]
BPA	PEC	35–280	12	Cu/g-C_3_N_4_	[71]
BPA	ECL	0.001–1	0.00003	C–g-C_3_N_4_	[72]
PCP	ECL	10^−8^–10^−4^	10^−8^	g-C_3_N_4/_graphene	[74]
HQ	CV	1000–320,000	300	AuNPs@g-C_3_N_4_	[75]
4-NT	CV	1000–10,000	100	ZnO/g-C_3_N_4_	[76]

BSO–gCN—barium stannate-graphitic carbon nitride; OX-g-C_3_N_4_—oxidized graphitic carbon nitride; PDDC–g-C_3_N_4_—poly(diallyl dimethylammonium chloride)-graphitic carbon nitride; ECL—electrochemiluminescence; C–g-C_3_N_4_—carboxylated graphitic carbon nitride; HQ—hydroquinone; CC—catechol; 4-NT-4—nitrotoluene.

**Table 3:** Recent reports on graphitic carbon nitride (g-C_3_N_4_)-based sensor for the detection of pharmaceuticals in water.

Analyte	Method	Linear Range (nM)	LOD (nM)	Materials	Ref
SMZ	Amperometry	20–623,000	5.78	g-C_3_N_4/_ZnO	[80]
CP	PEC	60–19,090	20	ND-g-C_3_N_4_	[81]
SMZ	PEC	0.5–80	0.1	g-C_3_N_4_ QDs	[82]
CTC	Fluorescence	20–1000	8	g-C_3_N_4_/MIS	[83]
PAR	DPV	2000–1680 × 10^3^	1000	PEDOT@g-C_3_N_4_	[84]

g-C_3_N_4_/MIS—graphitic carbon nitride molecular imprinted silica.

**Table 4:** Recent reports on the application of graphitic carbon nitride-based sensors for the detection of biomolecules.

Analyte	Method	Linear Range (mM)	LOD (mM)	Materials	Ref
Glucose	Fluorescence	0–10	42	CNQDTs/APBA	[89]
Glucose	Fluorescence	0.01–1	16	PBA/g-C_3_N_4_	[90]
Glucose	EIS	25–420	1.1	Co(OH)_2_–C_3_N_4_	[91]
Glucose	Amperometry	0.0006–2	300	C_3_N_4_/Fe_2_O_3_/Cu	[92]
Glucose	Amperometry	0.001–0.14	250	g-C_3_N_4_/Fe_3_O_4_	[93]
Dopamine	DPV	0.0005–8.5	60	CuO/g-C_3_N_4_	[94]
Dopamine	DPV	0.1–1	29,000	CaSt-g-C_3_N_4_	[100]
Dopamine	DPV	1.2 × 10^−5^–5	4	WP5@g-C_3_N_4_	[101]
H_2_O_2_	PEC	0.03–0.3	22	g-C_3_N_4_@Ag_2_O	[105]
H_2_O_2_	PEC	0.013–0.08	0.38	ZnO-g-C_3_N_4_	[106]
CEA	PEC	10^−7^–0.01	0.0001	g-C_3_N_4_–BiOCl	[109]
HER	PEC	5 × 10^−7^–10^−6^	0.00008	HCNT-AuNPs	[114]

g-CNQDTs/APBA—aminoboronic acid-functionalized graphitic carbon nitride quantum dots; PBA/g-C_3_N_4_—phenylboronic acid-graphitic carbon nitride; CaSt-g-C_3_N_4_—calcium stannate graphitic carbon nitride.
